# Clinical characteristics and immunotherapy in Chinese patients with Merkel cell carcinoma: a retrospective analysis

**DOI:** 10.3389/fonc.2026.1948121

**Published:** 2026-09-22

**Authors:** Ting Li, Sijia Ren, Jin Wang, Yingfan Zhao, Zhichao Liao, Zhiwu Ren, Ruwei Xing, Sheng Teng, Yun Yang, Jun Zhao, Weibin Xing, Jilong Yang

**Affiliations:** 1 Departments of Bone and Soft Tissue Tumor, Tianjin Medical University Cancer Institute and Hospital, National Clinical Research Center for Cancer, Tianjin, China; 2 National Clinical Research Center for Cancer, Key Laboratory of Cancer Prevention and Therapy, Tianjin’s Clinical Research Center for Cancer, State Key Laboratory of Druggability Evaluation and Systematic Translational Medicine, Key Laboratory of Molecular Cancer Epidemiology, Key Laboratory of Cancer Prevention and Therapy, Tianjin, China; 3 Tianjin Academy of Traditional Chinese Medicine Affiliated Hospital, Tianjin, China; 4 Second Clinical Medical College, Binzhou Medical University, Yantai, China

**Keywords:** anti-PD-1, clinical characteristics, immunotherapy, Merkel cell carcinoma, rare tumour

## Abstract

**Purpose:**

Merkel cell carcinoma (MCC) is a rare but highly aggressive cutaneous malignancy with limited epidemiological and therapeutic data in Asian populations. While immunotherapy works in Western patients, its efficacy in Chinese MCC patients remains unexplored.

**Methods:**

We retrospectively analyzed 40 histologically confirmed MCC cases treated at our institution between November 2011 to November 2024. Sixteen patients with complete records underwent detailed analysis of demographics, treatments, and responses.

**Results:**

The cohort included 25 female and 15 male patients (median age 71 years, range 25-91). Of the 16 patients with detailed information, 15 (94%) patients underwent extensive resection while 1 did not. Among surgical patients, 1 (7%) patient operative area received postoperative adjuvant radiotherapy, and 2 (13%) had additional lymph node dissection. Immunohistochemical analysis showed that out of 10 evaluable patients, 8 (80%) were MCPyV positive and 3 (30%) were PD-L1 positive. Among the patients receiving systemic treatment, four were administered Pembrolizumab at a dose of 200 mg every three weeks. Remarkably, three of these patients (75%) achieved a complete response (CR) by radiographic evaluation, with two of the treated specimens showed no residual malignant cells detected in post-treatment biopsy specimens. The treatment was well-tolerated, with no grade 3 or higher adverse events reported during the observation period.

**Conclusion:**

This study describes MCC characteristics in Chinese patients, with preliminary indications of female predominance and favorable immunotherapy responses. The findings highlight the need for standardized treatment approaches.

## Introduction

Merkel cell carcinoma (MCC) is a rare but highly aggressive neuroendocrine cutaneous malignancy originating from epidermal Merkel cells (1, 2). First described by Cyril Toker as “trabecular carcinoma of the skin” in 1972 (3), this neoplasm was later termed Merkel cell carcinoma. Similar to melanoma, MCC has the highest incidence in Australia and New Zealand, reaching 2.5 and 0.96 cases per 100,000 population, respectively (4). The disease predominantly affects elderly Caucasian males and exhibits two major etiological pathways: approximately 80% of cases are associated with Merkel cell polyomavirus (MCPyV) integration, while UV-induced mutations characterize most virus-negative tumors (5, 6). Clinically, MCC typically presents as a rapidly progressive, painless, flesh-colored to violaceous nodule or plaque on sun-exposed areas (7). These nodules or plaques enlarge significantly within weeks to months (7). The important diagnostic features of MCC can be summarized as AEIOU: Asymptomatic/lack of tenderness, Expanding rapidly ≤3 months, Immunosuppression, Older than age 50, and location on a UV-exposed site (7, 8).

Surgical management remains the cornerstone of treatment for localized MCC (9). Current NCCN guidelines (2024) recommend wide local excision with 1–2 cm margins to achieve adequate tumor clearance (10). However, despite optimal surgical resection, local recurrence rates remain substantial (25-40%), highlighting the aggressive nature of this malignancy (11, 12). Notably, evidence from a large retrospective analysis of 6,908 MCC cases demonstrated no survival benefit with adjuvant chemotherapy, supporting its omission in standard postoperative management (13). Therefore, patients without risk factors generally do not require adjuvant therapy after surgery. For patients with advanced disease, the main treatment for metastatic MCC before 2013 was chemotherapy, including platinum in combination with etoposide or cyclophosphamide in combination with doxorubicin, but with limited efficacy (9). The 5-year survival rate for patients with local lymph node metastasis was about 39-42%, and that for patients with distant metastatic disease was only less than 18% (14). Immune checkpoint inhibitors have since emerged as first-line therapy for metastatic MCC, demonstrating superior clinical outcomes in pivotal trials (15, 16). This transition reflects MCC’s immunogenic profile and has significantly improved management of advanced cases.

The status of MCPyV is closely related to the prognosis of MCC patients (17). Existing studies have shown that patients with MCPyV positivity usually have a better prognosis than those with MCPyV negativity (18). This difference is mainly due to the different carcinogenic mechanisms and immune microenvironment characteristics of MCPyV positive tumors: MCPyV positive tumors have fewer genetic mutations, and their carcinogenic process is mainly driven by viral oncogenes. At the same time, viral antigens can continuously stimulate the body’s immune response, forming: significant CD8+ T-cell infiltration within the tumor microenvironment (19, 20); while virus-negative tumors typically exhibit elevated tumor mutational burden (TMB) (21). Similar to observations in melanoma, there is no significant correlation between the expression level of PD-L1 protein and the response to anti-PD-1 therapy, therefore MCPyV positive status is considered an effective prognostic factor (17, 21). These differential immunogenic mechanisms driven by viral antigens or neoantigen load together form the biological basis for MCC’s excellent responsiveness to immune checkpoint inhibition (19).

In 2014, a multicenter, international, prospective, open-label, phase-II trial was conducted (22). A total of 88 patients were included in the study, aiming to evaluate the efficacy and safety of avelumab in advanced MCC that failed first-line treatment (22). This pivotal trial demonstrated an objective response rate (ORR) of 31.8% (95% CI 21.9-43.1), including 9% complete responses (CR), with durable responses observed in 82% of responders (23/28) at median 10.4-month follow-up (22). These results led to avelumab’s 2017 FDA approval as the first immunotherapy for MCC (23), marking a paradigm shift in treatment approaches. In addition to PD-L1 inhibitors, the PD-1 inhibitors Pembrolizumab and Retifanlimab as first-line treatments for advanced MCC was substantiated through two pivotal clinical trials (24). Keynote-017, a multicenter phase II study enrolled 50 MCC patients who did not receive systemic therapy and were treated with Pembrolizumab (2 mg/kg every 3 weeks) for 2 years (25). According to the research results, the ORR of Pembrolizumab was 56%. Treatment related adverse events of grade 3 or above occurred in 14 patients (14/50, 28%) (25). Similarly, the PODIUM-201 trial investigating Retifanlimab in 65 previously untreated patients showed a 52% ORR (95% CI 40-65), including an 18% complete response rate, with 76% of responders maintaining clinical benefit for at least 6 months (26), which supported its subsequent FDA approval in 2023 (24).

To date, there are limited data on the specific epidemiology and treatment-related aspects of MCC in China. A 2018 meta-analysis of Chinese cases (n=171; 1986-2017) revealed a distinct gender distribution (93 females vs 78 males) contrasting with Western cohorts where male predominance is typical (27). While immunotherapy has demonstrated efficacy in Caucasian populations, potential ethnic variations in treatment response remain unexplored. Genetic differences between Asian and Caucasian populations may influence both disease characteristics and therapeutic outcomes. This study aims to characterize the clinicopathological features and immunotherapy responses in our center’s MCC cohort (non-Caucasian) and provide comparative analysis with published Caucasian data.

## Materials & methods

### Patients

This retrospective cohort study analyzed 40 consecutive patients with histologically confirmed Merkel cell carcinoma who presented to Tianjin Medical University Cancer Hospital over a 13-year period (November 2011-November 2024). Using our institutional cancer registry, we identified all MCC cases through a comprehensive search of pathology databases using ICD-O-3 codes (8247/3) and histology reports. The cohort comprised two distinct groups: 20 patients who solely underwent pathological consultation without subsequent treatment at our institution, and 20 patients who received definitive treatment (**Figure 1**). After excluding 4 patients lost to follow-up, 16 patients with complete clinical data were included in the final analysis. The treatment cohort consisted of 12 patients managed with local therapies alone and 4 patients who received systemic immunotherapy. Among the 12 patients treated with local therapies alone, 11 had lesions anatomically suipathological complete response for wide local excision and were not considered for systemic therapy per multidisciplinary consensus. The remaining patient in this group was recommended immunotherapy but declined owing to financial burden. The 4 patients who received systemic immunotherapy were considered at high risk of progression (large tumor size, deep invasion, or suspected nodal involvement) after multidisciplinary evaluation and shared patient-physician decision-making.

**Figure 1:**
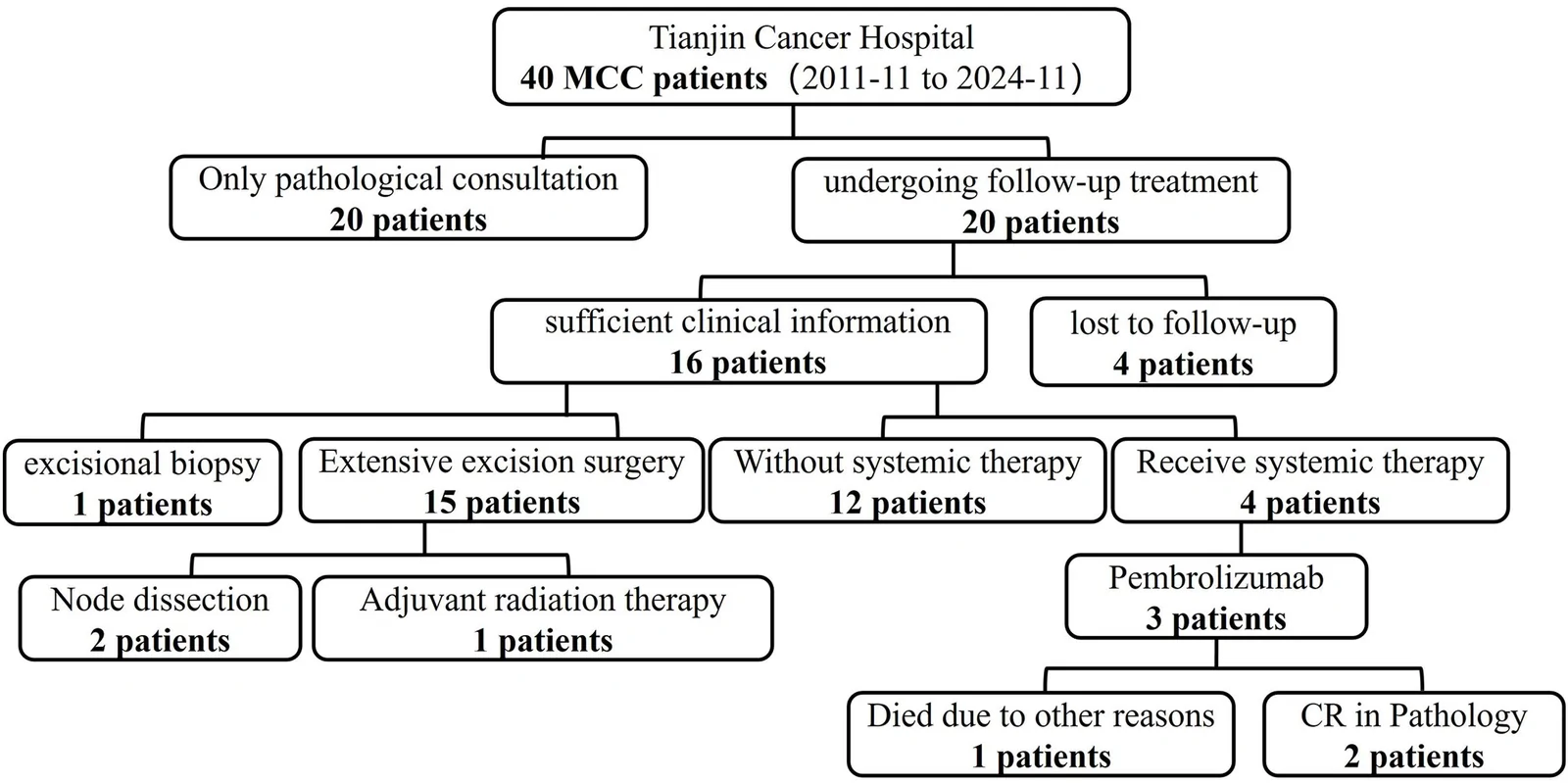
The flow charts of patient selection.

### Treatment methods

Fifteen patients underwent extensive resection of local lesions with a surgical margin of 2cm, and according to postoperative pathological examination, all patients achieved a negative surgical margin. Due to limitations in operating room conditions, none of the patients in this study underwent sentinel lymph node biopsy. Patients in the systemic therapy arm received intravenous pembrolizumab at a fixed dose of 200 mg administered over 30 minutes every three weeks, consistent with the FDA-approved dosing regimen for advanced MCC. Treatment response was assessed radiographically every 3 cycles according to RECIST v1.1 criteria. Safety monitoring included comprehensive laboratory tests and clinical evaluation before each treatment cycle. One patient who was treated with Pembrolizumab did not undergo efficacy evaluation after 4 cycles of treatment, and the patient died of acute myocardial infarction unrelated to treatment.

### Immunohistochemistry

Among all the patients, there were 10 patients with formalin-fixed, paraffin-embedded (FFPE) tumor specimens at our center. IHC was performed using rabbit antibodies against human PD-L1 (1:100; #13684, Cell Signaling Technology, USA). mouse antibodies against human CM2B4 was used as marker for MCPyV infection (200 µg/ml; sc-136172, Santa Cruz, USA). For each marker, staining intensity in tumor cells was scored as 0 to 2 (0= no staining, 1 = weak staining, and 2 = strong staining); the percentage of positive cells was calculated as 0: no staining, 1: <10%, 2: 10-30%, and 3: ≥30%, and the final score was determined by the sum of the staining intensity and positive percentage. The expression of PD-L1 was considered positive when ≥5% of tumor cells were positive. The tissue specimens were examined and scored by two pathologists.

### Follow-up schedule and methods

Patients are usually followed up every 3 months for the first 2 years of diagnosis, every 6 months for 3–5 years, and every year after 5 years. For patients undergoing systemic therapy, efficacy evaluation should be conducted every 3–4 cycles based on the RECIST v1.1 criteria. The follow-up items mainly include chest/abdominal CT, regional lymph node ultrasound, and local ultrasound in the surgical area.

### Ethics statement

The study was conducted in accordance with the principles outlined in the Declaration of Helsinki. This retrospective study was approved by the Ethics Committee of Tianjin Medical University Cancer Institute and Hospital (Approval No. bc20262181). All patients signed complete written informed consent at admission; The consent form states that the patient’s information and test results can be used for scientific research without compromising the patient’s privacy.

### Statistical analysis

Descriptive statistics and graphical representations were generated using GraphPad Prism 7 (GraphPad Software). Comparative analyses of clinical characteristics were performed using SPSS Statistics 23.0 (IBM Corporation). Survival analysis was conducted using Kaplan-Meier method with log-rank test for comparisons. All statistical analyses were performed using two-tailed tests with α=0.05 considered statistically significant.

## Results

### Patient characteristics

The clinical presentation of MCC in our cohort aligned with classic descriptions, manifesting predominantly as painless, rapidly growing pink-to-lavender nodules on sun-exposed areas (**Figures 2a-c**). Anatomic distribution analysis revealed: head and neck (9/16), the limbs (6/16) and rarely in the trunk (1/16) (**Table 1**). Notably, we observed a striking female predominance across our entire cohort (25 females vs 15 males, 63%) that persisted in the analyzed subgroup (11 females vs 5 males, 69%) (**Table 1**), consistent with previous Chinese reports but contrasting with Western data showing male predominance (27, 28).

**Figure 2:**
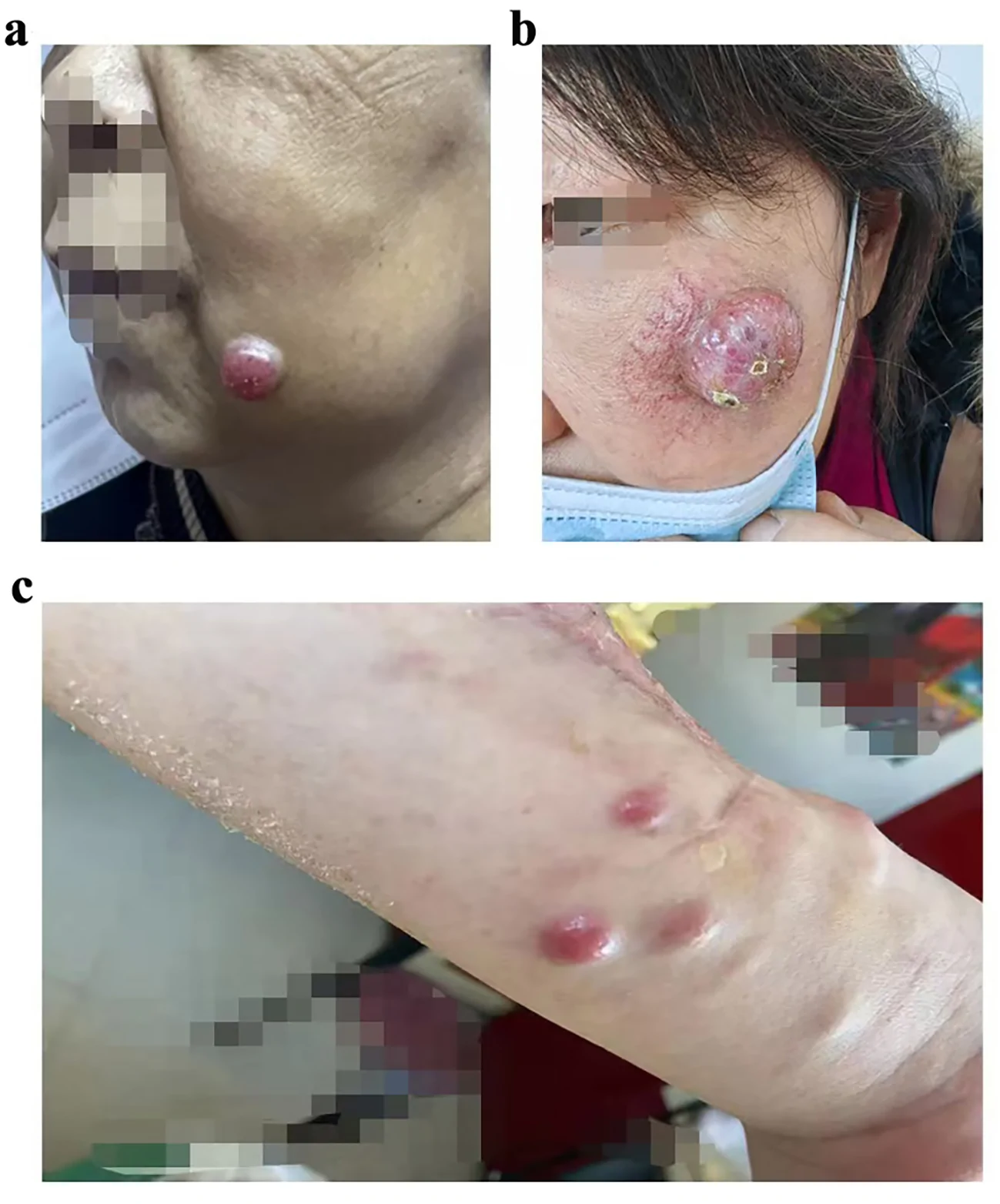
Common sites of Merkel cell carcinoma. **(a)** A pink nodule on the cheek; **(b)** an ulcerated lesion on the cheek; **(c)** multiple nodules on the forearm.

**Table 1:** Basic information, clinical characteristics and pathologic data of 16 patients with MCC.

Parameter	No. of patients (N = 16)
site	
head and neck	9
limbs	6
trunk	1
Gender	
male	5
female	11
Age	
≥60 years	14
<60 years	2
Tumor size	
≤ 2 cm	7
2 to 5cm	7
> 5cm	2
Deep tissue infiltration	0
Ulcers	
Yes	6
No	10
Lymph node	
N0	13
N1	3
N2	0
N3	0
Distant metastasis	
Yes	3
No	13
AJCC stage	
I	5
IIA	4
IIB	1
IIIA	3
IIIB	0
IV	3
Tumor location	
Primary site only	10
Lymph site only	0
Primary site and Lymph site	3
Visceral	3

In terms of the age of onset, consistent with other studies, the results of our study also suggest that the incidence of patients is increasing with age. Among all 40 patients, the median age was 71 years, and 31 (78%) patients were older than 60 years; Among the 16 patients who underwent follow-up treatment, the median age was 70.5 years old, of which 14 (88%) were older than 60 years old.

Among the 16 patients with sufficient clinical information (**Table 1**), the tumor size of most patients was less than 5cm (7 patients had tumors ≤ 2cm, and 7 patients had tumors between 2 and 5cm). Six patients’ tumors were accompanied by ulcers, and 10 patients’ tumors were without ulcers. There were 13 cases without lymph node metastasis and 3 cases with lymph node metastasis. There were 3 cases with distant metastasis and 13 cases without distant metastasis.

Within the limitations of our small sample size, these findings suggest several features of MCC in this Chinese cohort that may differ from those reported in Western populations, particularly regarding gender distribution and anatomic site predilection. The observed preponderance of head/neck lesions (57%) is consistent with a potential role of UV exposure in pathogenesis—a possibility that warrants further investigation even in Asian populations with generally higher skin pigmentation.

### Survival analysis: descriptive observations for MCC

Among the 16 patients with complete clinical data, median overall survival (OS) was not reached during the study period (**Supplementary Figure S1a**). The median follow-up duration for this cohort was 15.7 months (range, 2.8 to 112.1 months). Univariate Kaplan-Meier analysis was performed to explore potential associations between clinical characteristics and survival outcomes. As shown in **Supplementary Figure S1b, c, d**, patients with metastatic disease, head and neck tumors, or ulcerated lesions appeared to have poorer survival compared to their counterparts. However, due to the limited sample size and small number of events, no statistically significant prognostic factors could be identified.

### Merkel cell carcinoma and anti-PD-1 therapy

Of all the patients, only four received Pembrolizumab treatment (200mg every 3 weeks). Three patients (75%) completed a comprehensive treatment response assessment; One patient (25%) died of acute myocardial infarction before receiving radiological evaluation after four courses of drug treatment. Among the evaluated patients, the complete response (CR) rate was 100% (3/3) (**Table 2**); Persistent reactions were observed in all assessable cases.

**Table 2:** Efficacy of Pembrolizumab in 4 patients with MCC.

Parameter	No. of patients (N = 4)
CR	3
PR	0
SD	0
PD	0
No assessment	1
Objective response rate (ORR)	75%

In terms of safety, there were no reports of ≥ grade 3 treatment-related adverse events, and excellent treatment tolerance was observed in all dosing cycles. Regarding safety, no grade ≥ 3 treatment-related adverse events were recorded, and all patients tolerated pembrolizumab across treatment cycles. These observations from a small subgroup of four patients suggest that pembrolizumab is well-tolerated and may exert anti-tumor activity in some Chinese MCC patients. Nevertheless, definitive efficacy conclusions cannot be drawn given the limited sample size. Histopathological evaluation of two post-treatment specimens revealed no detectable residual malignant cells, which raises the possibility of long-term disease-free survival; however, extended follow-up data are required to verify this finding. While safety profiles appeared favorable in our cohort, direct cross-population safety comparison with Western MCC cohorts is constrained by the small sample.

### Immunohistochemical analysis of MCPyV status and PD-L1 expression

In non-Caucasian populations, viral infections, particularly MCPyV, are considered a major contributor to Merkel cell carcinogenesis. To preliminarily explore this within our small cohort, paraffin-embedded tumor specimens were available for immunohistochemical staining from 10 of the 16 patients with complete clinical data. CM2B4 staining was used as a surrogate marker for MCPyV infection. Among these 10 patients, 8 (80%) were positive (**Figure 3a**) and 2 (20%) were negative (**Figure 3b**). 

**Figure 3:**
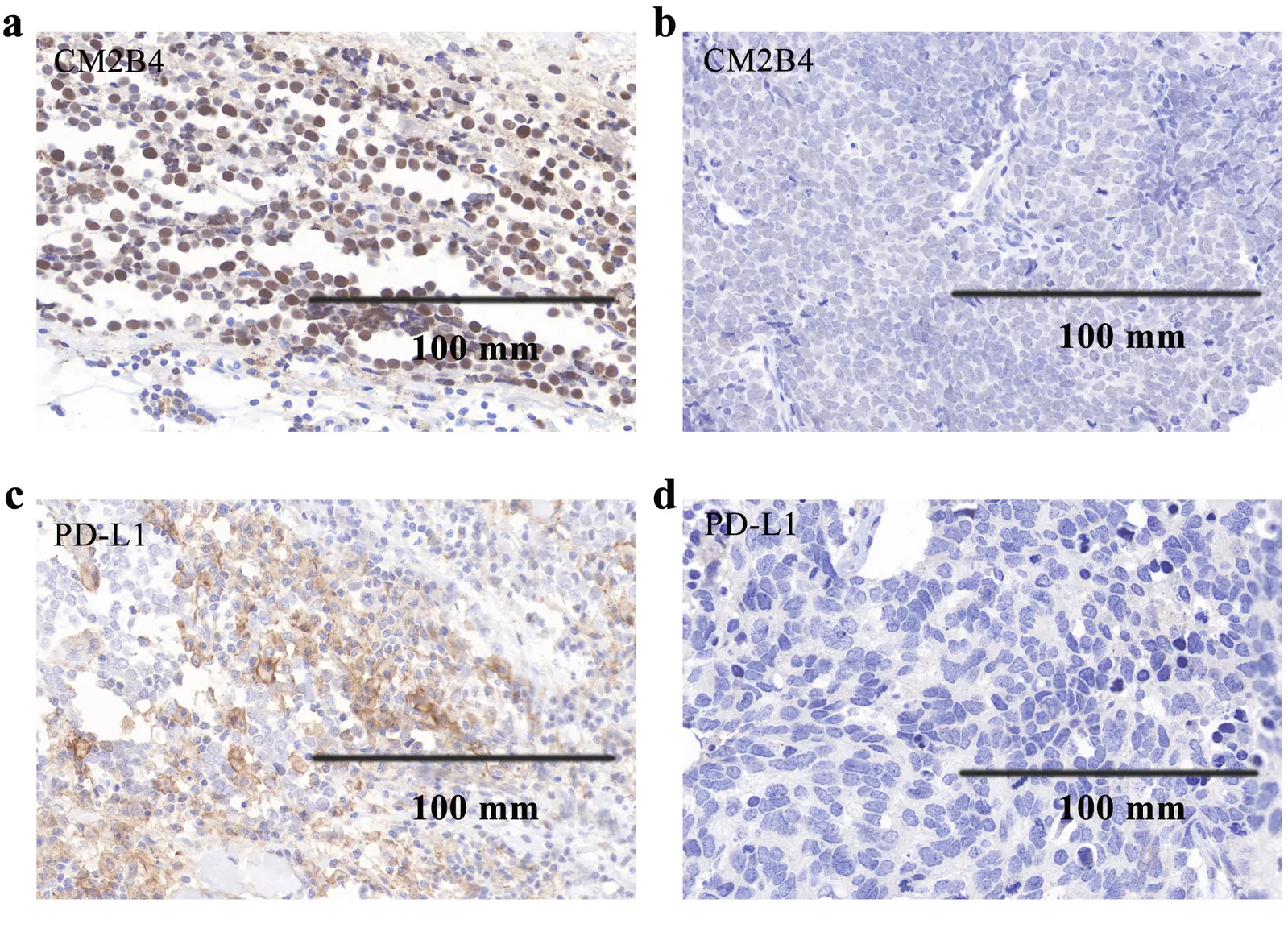
The expression of CM2B4 and PD-L1 in Merkel cell carcinoma tissue. **(a)** Positive expression of CM2B4 in the nucleus. **(b)** Negative expression of CM2B4 in the nucleus. **(c)** Positive expression of PD-L1 on the cell membrane. **(d)** Negative expression of PD-L1 on the cell membrane.

Among the four patients who received pembrolizumab, tissue was available for CM2B4 staining in three cases: two were MCPyV positive, both of whom treated specimens showed no residual malignant cells detected in post-treatment biopsy specimens, while the one MCPyV negative patient showed no response to immunotherapy.

Among the 10 assessable cases, PD-L1 expression was detected in 3 cases (30%) (**Figure 3c**) and negative in 7 cases (70%) (**Figure 3d**). Due to limited tissue availability, tumor mutational burden (TMB) could not be assessed, which represents a notable limitation in characterizing genomic correlates of immunotherapy response in this cohort. All findings should be interpreted with caution and require validation in larger, multicenter studies.

### Typical cases

A 90-year-old female visited Tianjin Medical University Cancer Hospital on January 3, 2024, five days after the excision of the left temporal skin tumor (**Figure 4a**). The patient found the left temporal skin tumor 5 years ago and did not pay attention to it at that time. Since then, the tumor slowly increased in size. In the last 3 months, the patient consciously felt that the tumor grew rapidly. Therefore, on December 30, 2023, she went to the local hospital for an excision of the left temporal skin tumor. Postoperative case consultation at Tianjin Medical University Cancer Hospital showed: left temporal cutaneous neuroendocrine carcinoma (**Figure 4b**). The characteristic immunohistochemical staining: CgA (+), Syn (+), CK20 (+) are shown in **Figures 4c–e**. It is also evident from the pathology results that the tumor cells have a fast proliferative capacity (Ki67 positivity rate of about 50%, **Figure 4f**).

**Figure 4:**
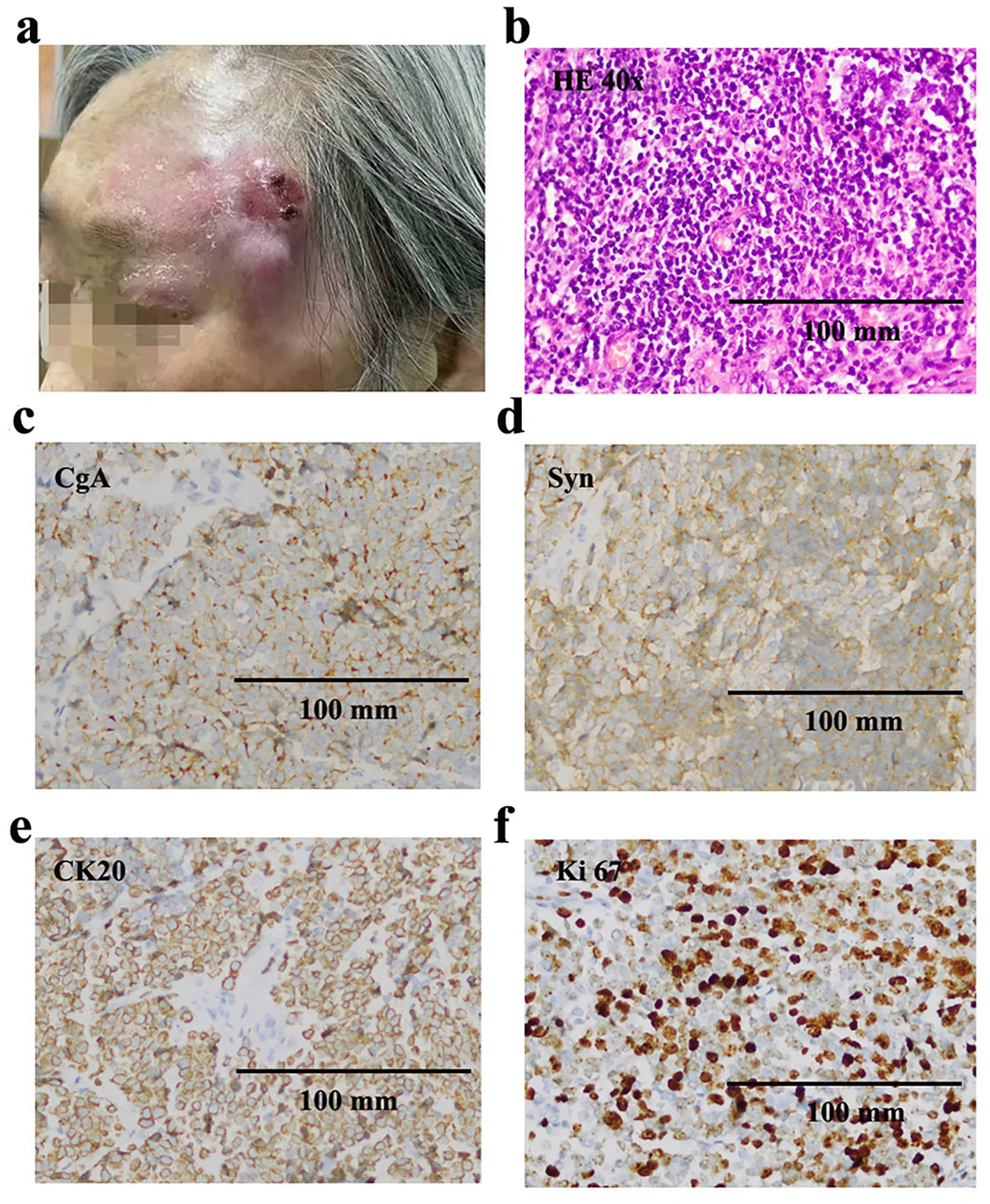
The patient’s appearance, HE staining images and characteristic immunohistochemical staining images. **(a)** Photographs of the patient’s appearance at the time of the first consultation; **(b)** 40x HE image; **(c)** Chromogranin A (CgA) (+); **(d)** Synaptophysin (Syn) (+). **(e)** Cytokeratin 20 (CK20) (+). **(f)** Nuclear protein Ki 67 positivity is about 50%.

After the diagnosis was confirmed, a systematic examination (including cervical lymph nodes and lung spiral CT) was first performed. The examination showed no obvious signs of malignancy or metastasis.

Considering that the tumor is located in the left temporal region, it was difficult to perform a wide resection without affecting the patient’s normal facial appearance. After the pathology was confirmed, the patient began systemic treatment with Pembrolizumab 200mg every 3 weeks on January 16, 2024. After 1 cycle of treatment, the tumor in the patient’s temporal region had clear boundaries (**Figure 5a**), followed by 4 cycles in which the area of affected skin in the patient’s temporal region became smaller and necrosis began to appear on the surface of the tumor (**Figure 5b**). With 6 cycles of anti-PD-1 treatment, the patient’s left temporal skin tumor was completely detached (**Figure 5c**).

**Figure 5:**
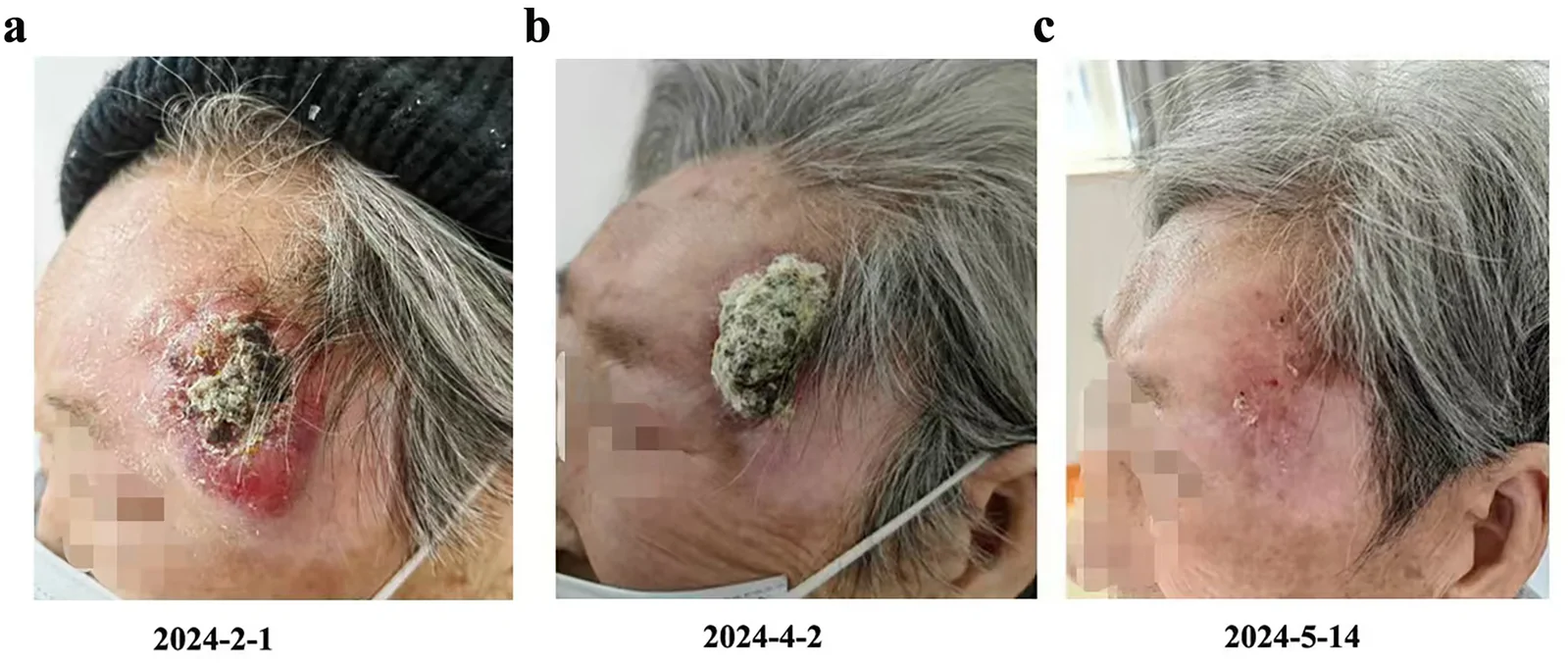
Changes of tumor during anti-PD-1 treatment. **(a)** After 1 cycle of treatment with anti-PD-1; **(b)** After 4 cycles of treatment with anti-PD-1; **(c)** After 6 cycles of treatment with anti-PD-1.

After two more cycles of consolidation treatment, the patient went to the Affiliated Hospital of Tianjin Academy of Traditional Chinese Medicine for dermoscopic examination (**Figures 6a, b**) and multiple-punch biopsy (**Figures 6c-f**) of the left temporal lesion area. As can be seen from the results, irregularly distributed yellowish-white fibrous cords were visible in the dermoscopic observation area, and linear blood vessels and pigmentation were visible at the edges of the lesion (**Figures 6a, b**). The pathologic findings also only suggested epidermal hyperkeratosis of the left frontal skin, focal liquefaction of basal cells, a few lymphocytes in the epidermis, and more lymphocytic infiltration in the superficial layer of the dermis (**Figures 6c, d**). Characteristic immunohistochemical staining showed negative expression of both Syn and CK20 (**Figures 6e, f**). No residual malignant cells were detected in post-treatment multiple-punch biopsy specimens of the tumor bed, and the treatment process was smooth without obvious drug-related adverse reactions.

**Figure 6:**
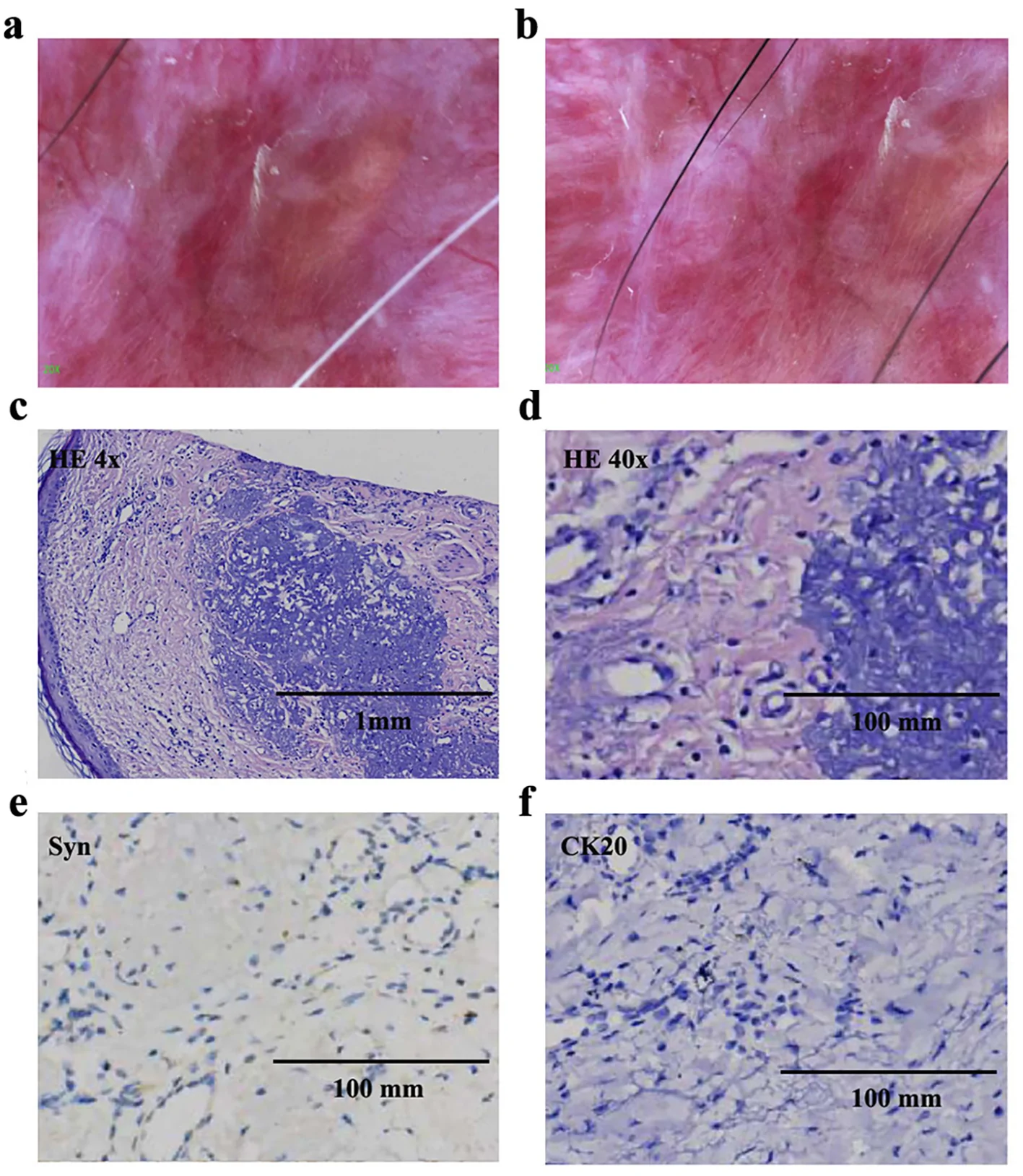
The dermoscopic images and pathological images of the tumor after 8 cycles of anti-PD-1 treatment. **(a, b)** Dermoscopic images after 8 cycles of anti-PD-1 treatment; **(c)** 4x HE image; **(d)** 40x HE staining showing the tumor bed with no residual malignancy identified. The dark-blue hematoxylin-stained areas represent reactive inflammatory infiltrates and fibrous granulation tissue; **(e)** Synaptophysin (Syn) (-); **(f)** Cytokeratin 20 (CK20) (-). Immunohistochemical images **(e–f)** are representative fields sampled from the same multiple-punch biopsy specimen, but different microscopic fields from panels **(c–d)**.

## Discussion

MCC is a rare neuroendocrine cutaneous malignancy with higher incidence rates observed in regions with greater ultraviolet (UV) radiation exposure (8). While the precise pathogenesis remains incompletely elucidated, current evidence supports a multifactorial etiology involving Merkel cell polyomavirus (MCPyV) infection, cumulative UV damage, and immunosuppression status as key contributing factors (29). Notably, emerging data suggest potential ethnic variations in MCC characteristics between Asian and Caucasian populations, likely attributable to differences in genetic predisposition and environmental exposures (30). Despite these recognized disparities, Asian countries currently lack both fundamental research and prospective clinical studies on MCC. Our study addressed this knowledge gap by conducting a comprehensive retrospective analysis of 40 histologically confirmed MCC cases from a single center in China over a period of 13 years. In this study, we conducted a detailed characterization of the baseline clinical pathological features of the entire cohort, and 16 patients with sufficient clinical information were analyzed further.

Our findings indicated several demographic characteristics of Merkel cell carcinoma (MCC) in this Chinese cohort that may differ from those reported in Western populations. Notably, we observed a female predominance (63%), which aligns with previous Chinese studies (27, 28), but contrasts with the male predominance typically reported in Caucasian populations (19). The reasons for this discrepancy remain unclear but could involve genetic background differences between Asian and Caucasian populations, as well as potential variations in lifestyle or environmental exposures. Given the limited cohort size, however, we cannot exclude the possibility that this observed sex distribution does not fully represent the broader Chinese MCC population. Larger, multicenter studies are needed to clarify this issue.

Similarly, some studies in Japan and South Korea also suggest that MCC patients are mainly women, while reports in Taiwan, China have observed that male patients dominate (31–35). This regional variation within Asia, if confirmed, may involve differences in genetic background, environmental exposures, or lifestyle factors—an area warranting further investigation. Our data are consistent with the established understanding that MCC primarily affects elderly individuals, with 78% of cases occurring in patients over 60 years (median age 71 years), likely reflecting age-related immunosenescence and cumulative carcinogenic exposures (36). Cross-population comparison reveals notable differences in MCC profiles. Western cohorts are characterized by male predominance, high proportion of head-neck primary tumors, and relatively high MCPyV-positive rates. By contrast, female preponderance has been reported in several mainland Chinese and East-Asian MCC cohorts, which is inconsistent with Western epidemiological patterns. Nevertheless, this observation should be interpreted cautiously, given the limited sample size and heterogeneity among available Asian retrospective series. Importantly, our real-world retrospective cohort cannot be directly matched to prospective Western immunotherapy registration trials, because of discrepancies in patient selection, baseline risk factors, and clinical practice patterns.

With the increasing age and the deterioration of immune status, all these are important factors that lead to the development of MCC (29). In addition, MCPyV is also one of the important causes of MCC (29). In our study, we performed CM2B4 testing on 10 patients with preserved tissue specimens to represent the infection status of the virus. According to our results, the majority of patients in our population are positive for viral infections (80%), which is consistent with literature reports suggesting that viral infections are the main driving factor in non-Caucasian populations (31–35). However, given the small number of cases tested, this finding should be considered preliminary and requires validation in larger, independent cohorts.

Recently, a research team in Taiwan published a multicenter Taiwan MCC cohort consisting of 38 cases, which identified clinical evidence of chronic arsenic poisoning in 5 patients (13%) (37), indicating that arsenic exposure may be the third pathogenic factor in this population, in addition to MCPyV and ultraviolet radiation (37). However, in the current cohort, we do not have any relevant studies on chronic arsenic poisoning. Nevertheless, this study extends our previous findings by providing detailed clinical and pathological features of MCPyV positive and MCPyV negative MCC, including unique histopathological features, Rb expression profiles, and survival outcomes.

The treatment strategy for Merkel cell carcinoma (MCC) should be tailored based on disease stage and patient characteristics. For localized lesions, wide surgical resection with 1–2 cm margins remains the standard of care, often supplemented by adjuvant radiotherapy in high-risk cases. However, systemic therapy becomes crucial for patients with unresectable primary tumors, regional progression, or distant metastases, where it can significantly improve both survival outcomes and quality of life. In the pre-immunotherapy era, chemotherapy regimens combining platinum agents with etoposide or cyclophosphamide-based protocols represented the mainstay of systemic treatment, yet demonstrated limited efficacy with response rates ranging from 20% to 61% in metastatic disease and often accompanied by substantial toxicity (38, 39).

In recent years, the advent of immune checkpoint inhibitors has revolutionized MCC management, establishing a new therapeutic paradigm characterized by superior response rates, more durable disease control, and improved tolerability profiles compared to traditional chemotherapy approaches. This evolution in treatment options underscores the importance of comprehensive staging and personalized therapeutic decision-making in MCC management (30). Since 2017, the FDA has approved multiple immune checkpoint inhibitors for MCC, beginning with avelumab for refractory disease, followed by pembrolizumab (2018) and Retifanlimab (2023) as first-line options (15, 21, 40, 41). These agents have demonstrated remarkable efficacy in advanced MCC, with objective response rates (ORR) exceeding 50%, significantly improving both survival outcomes and quality of life (21).

In our real-world setting with a small sample size, observations from four treated patients offer hypothesis-generating insights rather than confirmatory evidence. Among these, three achieved clinical benefit with no significant treatment-related adverse events reported. One patient died of an acute myocardial infarction unrelated to the treatment prior to response assessment. Given the very limited number of cases, these findings cannot confirm the safety or efficacy of anti-PD-1 therapy in Asian MCC patients, but they are broadly consistent with outcomes reported in larger global clinical trials.

However, challenges remain, as primary and acquired resistance mechanisms limit durable responses in some patients. Ongoing research is exploring novel therapeutic strategies, including growth inhibitor analogs, tyrosine kinase inhibitors, and other targeted agents (12), to expand treatment options for non-responders and improve outcomes across all MCC subgroups.

This study has several limitations. First, as a single-center retrospective analysis, it is subject to inherent selection and information biases. Second, the modest sample size (n=16) and limited number of events precluded multivariate analysis; consequently, no statistically significant prognostic factors could be identified. The observed associations regarding metastasis, tumor location, and ulceration are purely descriptive and require validation in larger cohorts. Third, sentinel lymph node biopsy was not routinely performed nodal status was primarily assessed by imaging, which may have led to under-staging and affected the accuracy of survival data. Fourth, due to sample limitations, we were unable to obtain paired tissue samples before treatment, thus making it impossible to evaluate the dynamic changes in PD-L1 expression and immune cell infiltration before and after immunotherapy. And although we assessed PD-L1 expression and MCPyV status in available specimens, tumor mutational burden (TMB) was not evaluated due to tissue limitations, precluding a comprehensive analysis of genomic determinants of immunotherapy response. Additionally, viral status remained undetermined for six patients, limiting our ability to fully characterize the etiological landscape of MCC in this population. Furthermore, this retrospective study lacks HLA-I and HLA-II genotyping data, since matched germline blood samples were not available. This limits our capacity to investigate potential links between HLA characteristics, MCPyV infection, PD-L1 expression, and immunotherapy outcomes. HLA profiling should be prioritized in future prospective MCC cohorts. Finally, the findings on immunotherapy efficacy, while promising, are derived from a very small subgroup (n=4) and should be considered preliminary. Despite these limitations, our study provides valuable real-world data on MCC in an under-represented Chinese population and highlights critical areas for future prospective research.

## Conclusion

In the Chinese population, as a rare skin endocrine cancer, the incidence of MCC gradually increases with age. The most common site is the head and neck. And unlike Caucasians, our MCC is more common in older female. For localized lesions, surgical wide excision is the most important treatment modality. In patients with locally advanced or metastatic disease, anti-PD-1 therapy showed antitumor activity in a small subgroup; however, these observations are preliminary and derived from a very limited number of cases. Larger, multicenter studies are urgently needed to validate these findings and to establish definitive treatment guidelines for MCC in Asian populations.

## Data Availability

The raw data supporting the conclusions of this article will be made available by the authors, without undue reservation.
