# Supplementary material for: Clinical characteristics and immunotherapy in Chinese patients with Merkel cell carcinoma: a retrospective analysis
Source: Front Oncol. 2026 Sep 22;16:1948121. doi: 10.3389/fonc.2026.1948121 (PMC13638394; doi:10.3389/fonc.2026.1948121)
Supplement: Supplementary file 1 [file DataSheet1.docx]

**Supplementary Figure 1. Survival analysis and prognostic factors in Chinese MCC patients.** a Kaplan-Meier curve showing overall survival (OS) for the entire cohort (n=16). b Metastatic status showed the strongest association with poor outcomes (p=0.074). c Head/neck tumors showed a negative prognostic trend (p=0.36). d Ulcerated lesions (n=6) correlated with reduced survival compared to non-ulcerated tumors (n=10) (p=0.396).
