# Supplementary material for: Clinical characteristics and immunotherapy in Chinese patients with Merkel cell carcinoma: a retrospective analysis
Source: Front Oncol. 2026 Sep 22;16:1948121. doi: 10.3389/fonc.2026.1948121 (PMC13638394; doi:10.3389/fonc.2026.1948121)
Supplement: Supplementary file 2 [file Table1.docx]

Supplementary Table 1. Comparison of clinicopathological characteristics and survival outcomes among mainland Chinese, Western, and other Asian Merkel cell carcinoma cohorts.

| Variable | Mainland Chinese cohorts (pooled retrospective series)^[1]^ | Western cohorts (US/Europe pooled real‑world data)^[2-4]^ | Other East‑Asian cohorts (Korea/Japan pooled)^[5, 6]^ |
| --- | --- | --- | --- |
| **Sex**, Male: Female | 0.69 : 1 | 1.8‑2.3 : 1 | 0.62‑0.77 :1 |
| **Median age** | 65.5 | 71‑76 | 66‑70 |
| **Primary tumor location** |  |  |  |
| Head and neck | 41 % | 60‑70 % | 32‑40 % |
| Extremities | 42 % | 20‑30 % | 45‑55 % |
| Trunk | 17 % | 8‑12 % | 5‑10 % |
| **MCPyV‑positive rate** | Limited pooled data | 70‑80 % | 69‑86% |
| **PD‑L1 positive expression** | Limited pooled data | 45‑65% | Limited pooled data |
| **5‑year overall survival** | Not well aggregated | 40‑52% | 65‑81% |
| **Immunotherapy objective response rate (real‑world)** | Small sample, limited data | 50‑65% | Small sample, limited data |

[1] SONG P I, LIANG H, WEI W Q, et al. The clinical profile of Merkel cell carcinoma in mainland China[J]. Int J Dermatol, 2012, 51(9):1054-1059.

[2] PEDERSEN E A, VERHAEGEN M E, JOSEPH M K, et al. Merkel cell carcinoma: updates in tumor biology, emerging therapies, and preclinical models[J]. Front Oncol, 2024, 14:1413793.

[3] D'ANGELO S P, LEBBé C, MORTIER L, et al. First-line avelumab in a cohort of 116 patients with metastatic Merkel cell carcinoma (JAVELIN Merkel 200): primary and biomarker analyses of a phase II study[J]. J Immunother Cancer, 2021, 9(7).

[4] SANTORO F, MALETTA F, PARENTE R, et al. Clinical-Pathological Evaluation and Prognostic Analysis of 228 Merkel Cell Carcinomas Focusing on Tumor-Infiltrating Lymphocytes, MCPYV Infection and ALK Expression[J]. Endocr Pathol, 2022, 33(2):289-303.

[5] NAKANO E, OGATA D, NAMIKAWA K, et al. Merkel cell carcinoma: a review of clinical management with focus on Asian patients[J]. Japanese Journal of Clinical Oncology, 2023, 53(8):673-679.

[6] MOON I J, NA H, CHO H S, et al. Clinicopathological characteristics and prognosis of Merkel cell carcinoma: a single-center retrospective study in Korea[J]. J Cancer Res Clin Oncol, 2023, 149(12):10065-10074.

Supplementary Table 2 Individual‑level data of other three patients receiving pembrolizumab.

| Patient ID | AJCC Stage | Prior treatment | MCPyV status | PD‑L1 status | Treatment duration (cycles) | Follow‑up duration (month) | Response duration (month) |
| --- | --- | --- | --- | --- | --- | --- | --- |
| 1 | ⅢA | no | positive | negative | 6 | 11.8 | 8.5 |
| 2 | ⅡA | no | negative | negative | 12 | 34.5 | 28.3 |
| 3 | ⅡB | no | - | - | 4 | 7.1 | - |
